# Multi-Class Classification of Breast Cancer Using 6B-Net with Deep Feature Fusion and Selection Method

**DOI:** 10.3390/jpm12050683

**Published:** 2022-04-26

**Authors:** Muhammad Junaid Umer, Muhammad Sharif, Seifedine Kadry, Abdullah Alharbi

**Affiliations:** 1 Department of Computer Science, COMSATS University Islamabad, Wah Campus, Rawalpindi 47000, Pakistan; junaid14841@gmail.com; 2 Department of Applied Data Science, Noroff University College, 4612 Kristiansand, Norway; skadry@gmail.com; 3 Department of Information Technology, College of Computers and Information Technology, Taif University, P.O. Box 11099, Taif 21944, Saudi Arabia; amharbi@tu.edu.sa

**Keywords:** deep features selection, machine learning, breast cancer, multi-class, fusion, 6B-Net, deep learning

## Abstract

Breast cancer has now overtaken lung cancer as the world’s most commonly diagnosed cancer, with thousands of new cases per year. Early detection and classification of breast cancer are necessary to overcome the death rate. Recently, many deep learning-based studies have been proposed for automatic diagnosis and classification of this deadly disease, using histopathology images. This study proposed a novel solution for multi-class breast cancer classification from histopathology images using deep learning. For this purpose, a novel 6B-Net deep CNN model, with feature fusion and selection mechanism, was developed for multi-class breast cancer classification. For the evaluation of the proposed method, two large, publicly available datasets, namely, BreaKHis, with eight classes containing 7909 images, and a breast cancer histopathology dataset, containing 3771 images of four classes, were used. The proposed method achieves a multi-class average accuracy of 94.20%, with a classification training time of 226 s in four classes of breast cancer, and a multi-class average accuracy of 90.10%, with a classification training time of 147 s in eight classes of breast cancer. The experimental outcomes show that the proposed method achieves the highest multi-class average accuracy for breast cancer classification, and hence, the proposed method can effectively be applied for early detection and classification of breast cancer to assist the pathologists in early and accurate diagnosis of breast cancer.

## 1. Introduction

Breast cancer is a widespread disease with a very high mortality rate, commonly present in middle-aged women all over the world. According to a cancer survey, breast cancer has been declared the second most common cause of demise after lung cancer among all types of cancer-related mortalities around the globe [1]. A recent survey of 36 cancers shows that the newly diagnosed cases of breast cancer are approaching a rate of 7.3%, with a death rate of 6.9%, which shows that breast cancer is the most prevalent cancerous disease with an excessive death rate [2]. The US breast cancer statistics show that there are expected to be 281,550 newly diagnosed cases of invasive cancer in 2021, and that 43,600 women will lose their lives due to this disease. Moreover, statistics show that approximately 30% of newly investigated cancer cases in American women in 2021 will be breast cancer [3]. An accurate and prior diagnosis of this deadly disease is highly desirable to overcome the death rate. It is estimated that approximately 90% of breast cancer patients may be cured and treated with an early and accurate diagnosis [4].

The initial diagnosis of breast cancer is carried out by physical examination and visual analysis of mammography and ultrasonic images. The final diagnosis of cancer is then confirmed using microscopic analysis of the extracted region after surgery. The pathologist makes the final diagnosis of breast cancer from a microscopic modality [5]. The manual diagnosis of this deadly disease is a laborious and complex task, due to a lack of standards, the need for expert pathologists, and it is also subjective. The diagnosis of the same patients may produce different results from different pathologists. According to a study, diagnosis conflict among different pathologists using biopsy samples is about 25% [6,7,8]. In most cases, breast cancer diagnosis is carried out with the help of histopathology images [9]. Diagnosis is being made easier with the advancement in computer technology machine learning (ML), and deep learning (DL) is being applied on a large scale to automate the manual diagnosis of different diseases [10,11]. Deep convolution neural networks (DCNN) produced promising results in medical diagnosis [12]. The high performance of DCNN in medical image analysis makes it possible to implement DCNN for breast cancer early diagnosis, by using histopathology analysis outcomes in the form of images. The DCNN utilized the different patches of whole slide images to detect the different categories of breast cancer, by using the majority voting scheme on the last layer of the network. Moreover, feature extraction using DCNN and ML algorithms for classification are also being implemented in the early detection of cancer [13]. Recently, many computer-aided solutions for the early and accurate detection of breast cancer have been proposed and successfully tested. Yassin et al. propose a study and discuss the different computer-aided diagnosis systems that utilize different imaging methods to accurately diagnose breast cancer. Their review suggests that a more efficient and accurate CAD system for breast cancer detection is highly desirous [14]. In another recent review, Fujiok et al. discuss the recent progress and challenges of breast cancer detection by using DL with the help of ultrasonography, and report that deep learning-based diagnosis of breast cancer is gaining increased attention, due to high performance and the research gap in terms of accuracy and model improvement [15]. For a more in-depth view of breast cancer segmentation and classification from histology images, interested readers are referred to the latest survey articles [16,17,18,19,20].

Most of the work in breast cancer classification attempted to solve the binary class classification of breast cancer. Very few studies have presented that try to solve multi-class breast cancer classification into four classes, and, in addition, the multi-class classification of breast cancer into eight classes is rarely discussed, due to the high resemblance of eight classes of breast tumors. Due to the high similarity in input images of different classes of breast cancer, it is a very challenging task to automatically classify breast cancer into eight classes. Most of the recent work in multi-class classification of breast cancer was carried out with simple transfer learning, or CNN models with fixed receptive fields of convolutional kernels that are unable to extract the discriminative features. To solve the multi-classification problem of breast cancer, this work proposed a 6B-Net with six branches implemented, each with different receptive fields to capture the most discriminative high-level features. For performance enhancement of the proposed method, feature fusion and selection are also implemented before the final classification task. Experimental results show that the proposed method achieves the best performance in terms of accuracy, and hence, the proposed method can effectively be applied for the early detection and classification of breast cancer to assist pathologists in the early and accurate diagnosis of breast cancer. 

## 2. Material and Methods

**Figure 1:**
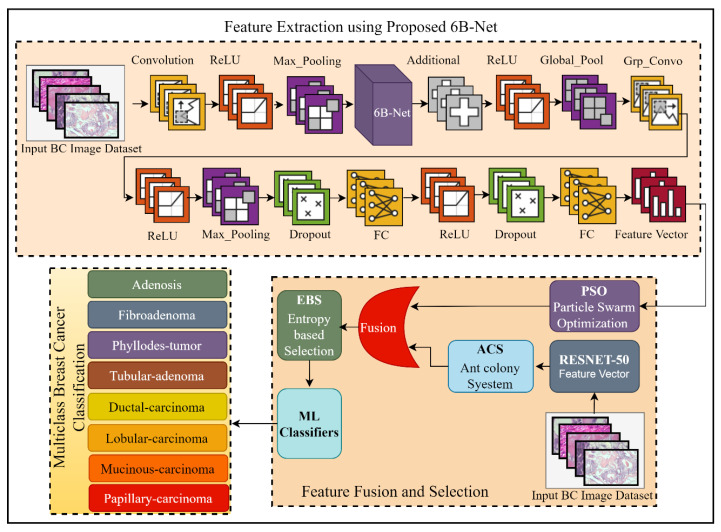
The framework of the proposed fusion and selection-based method of breast cancer multi-class classification. The feature extraction using the proposed 6B-Net block represents the feature extraction process; feature fusion and selection block represent the proposed feature fusion and selection process; and finally, the classification block represents the eight classes of breast cancer. Here BC is representing breast cancer, FC is representing the fully connected layer, and ReLU is the rectified linear unit.

The proposed DL-based method for breast cancer multi-class classification is presented in Figure 1. Breast cancer is a widely spread, fatal disease, and to overcome the mortality rate of this disease, an early and effective diagnosis is highly desirable. In this regard, this research work proposed a DL-based automated solution for multi-class breast classification. The proposed technique was comprised of three steps. Initially, a 35-layer deep CNN model with one concurrent processing block was introduced. The proposed network was first pre-trained using a third-party CIFAR-100 dataset [21] for feature learning. After the successful pre-training of the proposed model, this model was further utilized as a feature extractor for the breast cancer multi-class classification problem. After the feature extraction phase, the extracted feature vector was passed as input to the PSO feature selection algorithm for the best feature selection. In the feature extraction phase, a feature vector was also extracted by using pre-trained RESNET-50 [22], and a feature selection method of ACS was applied to this feature vector for feature selection. To enhance the performance of breast cancer classification, the outputs of the proposed 6B-Net model selected vector and RESNET-50 selected vector were fused serially. After serial feature fusion, a feature selection method of EBS was applied before classification. Finally, for classifying breast cancer into eight different classes, diverse ML algorithms were employed. The complete detail of the proposed CNN model and the selection process is presented in the next two subsections of the article.

### 2.1. Proposed 6B-Net CNN Model

**Figure 2:**
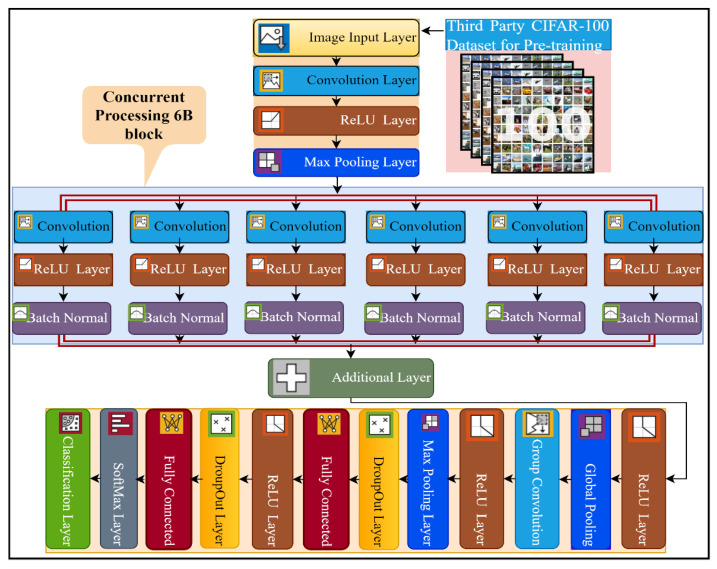
The pre-training and layer architecture diagram of the proposed 6B-Net deep CNN model.

This study proposed a novel six-branch deep CNN model for multi-class breast cancer classification. The details of the layers of the proposed network architecture are presented in Figure 2, and the complete implementation detail of every layer, with the number of kernels and filter size, is presented in Table S1. The proposed model consists of a total of 35 layers and 39 connections. The input size of the 6B-Net CNN model is 227 × 227 × 3, and it takes three-channel RGB images as input. Subsequently, the next 2D convolution layer was implemented with 128 kernels, of size 9 × 9. The purpose of the convolution layer is the extraction of features from the input image. The mathematical implementation of the convolution layer is presented in Equation (1), where $img_{con}$ represents the image after the convolution step, $fil_{st}^{sz}$ shows the filter, ‘sz’ represents the size of the filter (9 × 9), and ‘*st*’ represents the stride size, which in this case is 2. The first convolution layer was implemented with the same padding and dilation factor of 1,1. After the first convolution layer, the next activation layer was implemented with the ReLU. The purpose of the activation layer is the transformation of the weighted sum from input to output in the form activation function. The mathematical function of the activation function is presented in Equation (2). The third layer in the proposed network is the max-pooling layer, implemented with a window size of (5,5), stride size of 2, and the same padding strategy. The purpose of using the max-pooling layer is the downsampling of the input features, by only choosing the most prominent feature from the pooling window. The mathematical formulation of this layer is presented in Equation (3), where $MAX_{st}^{w}$ denotes the max-pooling function, ‘*w*’ represents the window size, and ‘*st*’ represents the stride size. This max-pooling window was convolved through the input images by selecting the maximum value from the window size for further processing.

(1) $$img_{con}=\sum_{-k}^{k}fil_{st}^{sz}\;*\left(img_{input}\;\cdot Weight\right)+bias$$

(2) $$\sigma_{ReLU}=f\left(img_{con}\right)=\{\begin{array}{c} 0,\;\;img_{con}<0\\ 1,\;\;img_{con}\geq 0 \end{array}\;$$

(3) $$img_{\max\_p}=\;MAX_{st}^{w}*\;img_{input}$$

In the next step, the proposed 6B-Net deep network introduced a novel block 6B, which contained six branches with concurrent processing. Each branch contained a convolution layer, a ReLU layer, and a batch normalization layer. The purpose of using the batch normalization layer was to normalize the data at the batch level for speeding up the training process of the model. The main difference between the six branches was the size of the convolutional kernel. To extract the high-level image feature, the filter size of each convolution layer was reduced gradually, from 13 × 13 to 3 × 3 in every concurrent branch of the proposed model. The number of convolutional kernels in each concurrent branch was kept at 96. Each branch was a combination of three layers, including convolution with different filter sizes, an activation function, and a batch normalization layer. The branch normalization was applied to every mini-batch of whole data in every epoch during the training process. The mathematics behind the batch normalization layer can be seen in Equations (4)–(6). In the batch normalization layer, mean ${\bar{M}}_{mini\_b}$ of the mini-batch was calculated using Equation (4), and then the variance $va{r^{2}}_{mini\_b}$ of the images were computed by utilizing Equation (5). In the last step for this layer, batch normalization ${\hat{B}}_{nor}$ was carried out using Equation (6), where γ and β are the learnable parameters. The main contribution of the proposed model is the concurrent processing block that mainly consists of six branches, each with different kernel sizes and an equal number of filters.

(4) $${\bar{M}}_{mini\_b}=\frac{1}{k}\overset{k}{\underset{1}{\sum }}img_{input}$$

(5) $$va{r^{2}}_{mini\_b}=\frac{1}{k}\overset{k}{\underset{1}{\sum }}{\left(img_{input}-\;{\bar{M}}_{mini\_b}\right)}^{2}$$

(6) $${\hat{B}}_{nor}=\;\frac{img_{input}-{\bar{M}}_{mini\_b}}{\sqrt{va{r^{2}}_{mini\_b}+\varepsilon }}\;\;\;\overset{yields}{\to }\;\;\gamma \;{\hat{B}}_{nor}+\beta$$

The mathematical formulation of six concurrent branches is presented in Equation (7). The parameter ${ℬ}_{i}$ represents the branch number, ‘*i*’ represents 1 to 6 branches, $fil_{2,2}^{ixj}$ shows the filter size with the stride of [2, 2], and *I* × *j* represents the filer sizes of six concurrent branches as 3 × 3, 5 × 5, 7 × 7, 9 × 9, 11 × 11, and 13 × 13, respectively. The $\sigma_{ReLUi}$ represents the activation function of each branch *i*, and ${\hat{B}}_{nori}$ represents the batch normalization layer for each branch *i*.

(7) $${ℬ}_{i}=\left(\left(\left(\;\;fil_{2,2}^{ixj}*\left(img_{input}\;\cdot Weight\right)+bias\right)\sigma_{ReLUi}\right)\;{\hat{B}}_{nori}\right)$$

After concurrent processing of six branches, an additional layer was implemented for the further feature learning process. In the next step, an activation layer with ReLU was implemented before the global pooling layer. In the global max-pooling layer, pooling was carried out globally, with a filter depth of 1 × 1 × 96. The working principle of global max-pooling is the same as max-pooling, but instead of taking values from the pool window, it takes the max value from the input image-sized window. After that, a grouped convolution layer was implemented, with a filter depth of 1 × 1 × 9216 and a stride of [1, 1]. In the next stage, the ReLU activation layer was implemented before the max-pooling layer. A max-pooling layer with a window size 5 × 5 was implemented with stride [1, 1] and the same padding. Subsequently, a 50% dropout layer was implemented, which ensures that every neuron has a 50% probability of being activated in the subsequent layer. After this layer, an FC layer with a size of 4096 was implemented, before the activation layer. Following this, a 50% dropout layer was implemented before the 2nd FC layer. In the last step, a softmax layer was implemented above the classification layer for a multinomial probability distribution. The mathematical form of this layer is presented in Equation (8), where $SoftMax\left(input_{i}\right)$ shows the softmax function that is taking the input feature I, $e^{input_{i}}$ is utilized to standardize the input data that produces a high value for positive input and a very small value for negative input, $\sum_{j=1}^{k}input_{j}$ represents the probability normalization term to set the output in the zero and one range, and k represents the number of classes.

(8) $$SoftMax\left(input_{i}\right)=\frac{e^{input_{i}}}{\sum_{j=1}^{k}input_{j}}$$

### 2.2. Feature Fusion and Selection

**Figure 3:**
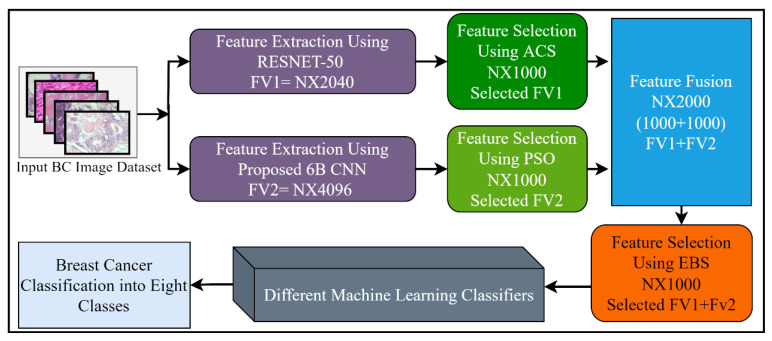
The proposed feature selection and fusion strategy with details of the number of features.

Feature selection (FS) is a method of choosing the most relevant and best features from the input feature vector, in order to enhance the classification accuracy in machine learning. Generally, features extracted from the CNN models, or by using other feature extraction methods, contain redundant information that misleads the classifiers trying to accurately classify the problem. A large feature set may also be a burden for further processing such as segmentation. Different FS techniques have been proposed to tackle these issues. In this study, three different well-known feature selection methods including ant colony system (ACS), particle swarm optimization (PSO), and entropy-based selection (EBS) were applied for the best feature selection. Furthermore, the outputs of these feature selection methods were combined serially, by using the feature fusion technique for performance enhancement. After the fusion of the 6B-Net and RESNET-50 feature vectors, the best features were chosen using the EBS algorithm for further multi-class classification of breast cancer disease. The proposed feature selection mechanism is presented in Figure 3. The working principle of these three algorithms is explained in the next three paragraphs. 

In the initial step of the FS strategy, the PSO algorithm was implemented as a selection method. The feature vector extracted from the proposed 6B-Net was passed as input to the PSO algorithm. PSO is a biologically inspired optimization method that works based on the collective behavior of bird flocks, and was first proposed by Kennedy and Eberhart [23]. In PSO, a set of solutions or particles known as the population are represented by a point in multidimensional space. In searching for the optimal solution, every particle flies through the search space, based on its flying memory and its neighbor particle experience. Each particle updates its parameters to improve the search space, based on the previous best and global best. The moving velocity that is updated according to the objective function in the next iteration is presented in Equation (9).

(9) $$Vel_{j}^{i+1}=Wt_{iner}\;\cdot \;Vel_{j}^{i+1}+a_{1}rm_{1}\left(pre_{best_{j}^{i}}-pos_{j}^{i}\right)+a_{2}rm_{2}\left(glob_{best_{d}^{i}}-pos_{j}^{i}\right)$$

Here, $Vel_{j}^{i+1}$ represents the velocity of the *j*th particle in *i* + 1 iteration, $Wt_{iner}$ denotes the inertial weight, $a_{1}\;\mathrm{and}\;a_{2}$ represent the acceleration constant, $pre_{best_{j}^{i}}$ represents the previous position of the particle, $pos_{j}^{i}$ denotes the article position in *d* dimensional search space, $glob_{best_{d}^{i}}$ shows the global best position of the particles, and $rm_{1}\;\mathrm{and}\;rm_{2}$ represent the random values between zero and one. The position updating equation of the *i*th particle is presented in Equation (10).

(10) $$pos_{j}^{i+1}=pos_{j}^{i}+Vel_{j}^{i+1}$$

A binary PSO algorithm is needed to utilize the power of PSO for feature selection, and was achieved by applying the sigmoid transformation. The purpose of this transformation was the controlling of velocity range between zero and one that is represented mathematically in Equation (11).

(11) $$\nabla pos_{j}^{i+1}=\frac{1}{1+e^{\left(-Vel_{j}^{i+1}\right)}}$$

The position updating of each particle was carried out based on the comparison of $\nabla pos_{j}^{i+1}$ of with $vec_{d}$, which is the d-dimensional vector that generally contains the uniform random values between zero and one, as represented in Equation (12).

(12) $$pos_{j}^{i+1}=f\left(x\right)=\{\begin{array}{c} 0,\;\;\nabla pos_{j}^{i+1}<vec_{d}\\ 1,\;\;\nabla pos_{j}^{i+1}\geq vec_{d} \end{array}$$

In the second step of feature selection, ACS [24] was applied as the best feature selection. The FV extracted from the pre-trained RESNET-50 CNN was passed as input to the ACS algorithm. The ACS selection algorithm is the derived version of the ant colony optimization algorithm that works based on the food search strategy of ants, in which the food path is updated based on pheromones. The probability of including the feature ‘*f*’ into the solution set by ant ‘*a*’ at any time *t* is calculated by Equation (13), where $s^{a}$ represents the feasible feature set that can be added by ant a, $\Gamma_{f}$ represents the pheromone of the feature, $\xi_{f}$ shows the heuristic function, and α and β represent the pheromone weight and heuristic function value, respectively.

(13) $$prob_{f}^{a}\left(t\right)=\{\begin{array}{c} \frac{{\left(\Gamma_{f}\left(t\right)\right)}^{\alpha }\;\cdot \;{\left(\xi_{f}\right)}^{\beta }}{\sum_{m\;\epsilon \;s^{a}}{\left(\Gamma_{m}\left(t\right)\right)}^{\alpha }\;\cdot \;{\left(\xi_{m}\right)}^{\beta }}\;\;if\;f\epsilon \;s^{a}\\ 0,\;\;\;\;\;\;\;\;\;\;\;\;\;\;\;\;\;\;\;\;\;\;\;\;\;otherwise \end{array}$$

In the third step of feature selection, the EBS selection method was applied [25]. Entropy-based FS is generally practiced in characterizing the input feature set for best selection. In this work, to compute the entropy of the input feature vector *FV* if we have m number of features, *FV* = {$\psi_{1},\;\psi_{2}\ldots \;\psi_{m}$}, and the probability of occurrence of each element is presented by the $\mathrm{Pr}\left(\psi_{j}\right)$, then the entropy of the feature vector is calculated with the help of Equation (14).

(14) $$Entropy\;\left(FV\right)=\overset{m}{\underset{j=1}{\sum }}\mathrm{Pr}\left(\psi_{j}\right)\cdot \log_{2}\mathrm{Pr}\left(\psi_{j}\right)$$

In the final step of feature selection, the two selected feature vectors extracted from 6B-Net and RESNET-50 were fused horizontally by using the feature fusion method, and the top features were selected by using *EBS*. The final selected vector was then passed as input to different ML algorithms for multi-class breast cancer classification. The feature-level fusion process of the three selection algorithms is presented in Equation (15), where, FSV represents the final selected vector, $F_{PSO}$ represents the *PSO* based selected vector, $F_{ACS}$ shows the *ACS* based selected vector, and $F_{EBT}$ represents the EBS-based selected vector.

(15) $$FSV=F_{EBS}\left(\left(F_{PSO}+F_{ACS}\right)\right)$$

### 2.3. Datasets

In this work, for the training and testing of the proposed method, two publicly available breast cancer multi-class classification datasets were utilized. The BreaKHis dataset was used as dataset_1, and the breast cancer pathology dataset was used as dataset_2. For experimental results, the ratio of training and testing images was set as 70:30 respectively. The ethical issues are not applicable in this work, as we used publicly available datasets for research purposes only.

- The first dataset, namely, BreaKHis, contains the eight classes of breast cancer and is available at [26]. The dataset is available with complete information about each class. All the dataset images were generated from H & E sustained breast cancer biopsies. The dataset was compiled at several exaggeration scales including 40×, 100×, 200×, and 400×, with a total number of 7909 images. The dataset was collected from 82 patients. All the images in the database are three-channel RGB images in PNG format. The file names include information about the malignancy type and subtype, and information about the dataset is included in the dataset directory.
- The second dataset, namely, the breast cancer pathology dataset of breast cancer pathology images with four classes is presented in [27], and is publicly available. This dataset is the high-resolution, publicly available dataset with four categories of breast cancer. The images in every class have a resolution of 2048 × 1536, and all the dataset is available in TIFF format. This dataset consists of 3771 breast pathology images. The samples images from this public breast pathology archive are presented in Figure S1.

## 3. Results

The detailed results of the proposed method for multi-class breast cancer classification are presented in this section. To demonstrate the robustness of the proposed fusion-based method of breast cancer classification, different experiments were performed using the two publicly available multi-class breast cancer datasets, with four and eight classes. The experiments were performed using 3-fold, 5-fold, and 10-fold cross-validation procedures. The training and testing ratio was selected as 70:30. All the experiments were performed using MATLAB 2019 as a simulation tool, on a core I7 personnel computer with 20 GB of RAM. The experimental results based on utilizing two multi-class breast cancer datasets are presented in the next two subsections.

### 3.1. Experimental Results on BreaKHis Dataset

In this section, the experimental outcomes of the proposed method using dataset_1 are presented in detail. Dataset_1, namely, BreaKHis, is a publicly available multi-class breast cancer dataset, with eight classes of breast tumor. The complete detail of the dataset is presented in the dataset section of this article. In this experiment, the results are computed using four configurations. In the first configuration, the results are computed with a pre-trained RESNET-50 deep model. The concept of TL is implemented to extract the deep features from the FC layer fc1000, and the extracted feature vector is represented by FV1. Finally, for the classification of eight breast cancer classes, two machine learning algorithms, ensemble subspace KNN (ESKNN) and ensemble subspace discriminant (ESD), are utilized. In the second configuration, the results are computed with the same procedure, using our proposed 6B deep CNN model. The feature vector generated in this step is presented by FV2. In the third configuration, the results are computed by applying feature selection methods. In this step, an FS method of ACS is implemented at FV1 before classification, and an FS method of PSO is implemented at FV2, before inputting it to the ML classifiers. In the fourth configuration, the results are computed using the horizontal feature fusion of FV1 and FV2. As a result of this step, a new fused feature vector is generated by the fusion of FV1 and FV2. In the second step of this configuration, an entropy-based feature selection method is applied at the fused vector before classification. The training time for model training and classification accuracy in percent, using the 10-fold method, are presented in Table 1.

The highest multi-class accuracy of 90.10% is achieved using the ESKNN classifier, with a training time of 147 s, while the ESD classifier produces 75.60% accuracy with a training time of 298 s. The experimental results using the five-fold method are presented in Table 2.

The experimental results using the three-fold cross-validation method are given in Table 3. The results present that the highest accuracy is achieved with the 10-fold method, while the lowest multi-class average accuracy is reported in the 3-fold method. From Table 1, Table 2 and Table 3, it is concluded that the lowest training time is observed with three-fold validation.

**Figure 4:**
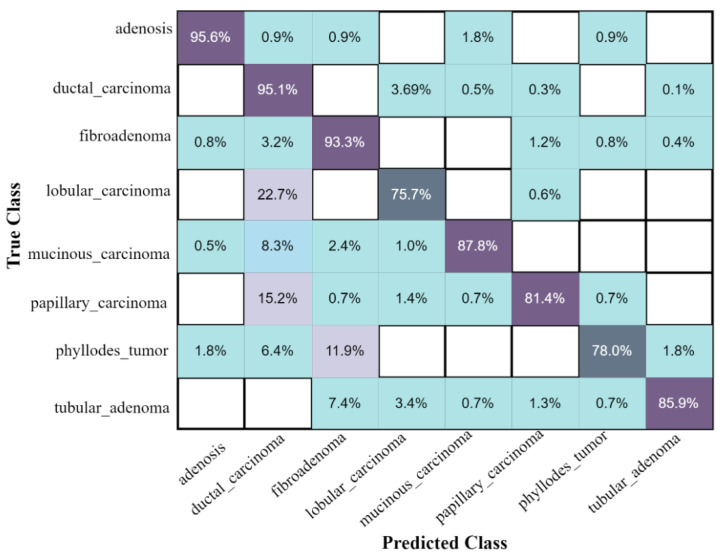
The confusion matrix results of the proposed method on the BreaKHis dataset, with ESKNN classifier using the 10-fold validation.

**Figure 5:**
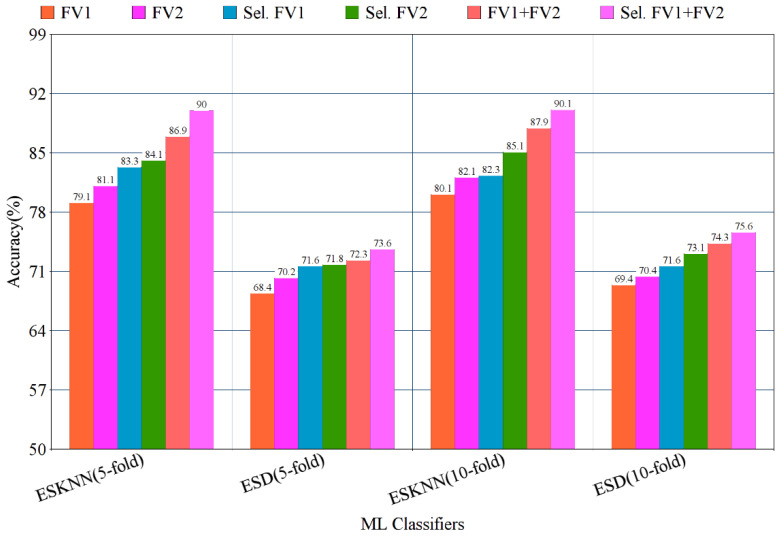
Bar graph representation of results using the proposed method on BreaKHis dataset, with five- and ten-fold validation.

The confusion matrix of eight classes of breast cancer with a ten-fold method, using the ESKNN classifier is presented in Figure 4. A comparison of the proposed feature fusion and selection-based method with the recent method of eight-class classification of breast tumors is presented in Table S2. Albashish et al. [28] utilize the pre-trained VGG-16 model for multi-class classification, and report an accuracy of 89.83%. In another work, Karthiga and Narasimhan [29] propose the deep CNN and transfer learning-based solution, with an accuracy of 89.29%. Rao PMM et al. [30] present the ensemble of the pre-trained CNNs, and report the highest accuracy of 89.00% with eight classes. Bardou et al. [31] propose the solution for the same task with classical and deep CNN models, and attain an eight-class classification accuracy of 88.23%. The proposed fusion-based method achieves the highest eight-class classification accuracy of 90.10%. For a more in-depth view of the presented results of the 10-fold method, a bar graph comparison is presented in Figure 5.

### 3.2. Experimental Results on Breast Cancer Pathology Dataset

In the second experiment, the results are computed using dataset_2, which contains high-resolution images of four categories of breast tumors. The complete detail of dataset_2 is presented in the dataset section of this article. In this experiment, after the feature extraction, fusion, and selection steps, six different ML learning classifiers are utilized for breast cancer multi-class classification. The experimental outcomes from applying a ten-fold procedure are tabulated in Table 4. The highest average accuracy of 94.20% is achieved using the ESD classifier, while the lowest accuracy of 83.70% is achieved using the EBT ensemble boosted tree classifier. The SVM support vector machine classifiers, with different kernels including cubic CSVM, quadratic QSVM, and linear LSVM, produce 85.62%, 86.82%, and 86.11% accuracy, respectively. The ESKNN classifier achieves an accuracy of 84.50%. 

In the second experiment, dataset_2 results are computed using a five-fold validation method. Experimental results using a five-fold cross-validation method are presented in Table 5. In the case of five-fold validation, the highest accuracy of 93.60% is achieved using the ESD classifier, while the lowest accuracy of 83.00% is achieved using the EBT classifier. All the results are computed using different configurations, based on the simple transfer learning of RESNET-50, and the proposed 6B-CNN model that produces FVI and FV2. Next, the results are computed at the selected feature vector, by applying ACS at FV1 and the selected feature by applying PSO at FV2. In the next configuration, the results are computed by applying horizontal feature fusion at both selected vectors FV1 and FV2. Finally, the EBS selection method is applied to the fused vector to compute the results. The experimental outcomes show that the highest accuracy is produced with the EBS selection after the fusion step. In all the results, the average accuracy and training time are recorded and reported in tabular form. 

**Figure 6:**
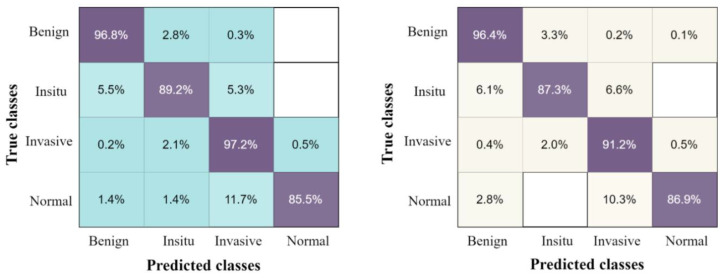
The confusion matrix results of the proposed method on breast cancer pathology dataset, with ESD classifier using the ten-fold method on the left, and five-fold method on the right.

The confusion matrix of the proposed results using four classes of breast tumors, with the highest accuracy classifier ESD and by applying five-fold and ten-fold methods, are presented in Figure 6.

The accuracy comparison of the proposed fusion-based method of four-class classification with existing work is presented in Table S3, which shows that the proposed method achieves the highest accuracy of 94.20% in four-class breast cancer classification.

## 4. Discussion

Breast cancer is a common and deadly disease that can be effectively cured by early detection and proper treatment. To perform an early and accurate diagnosis of this disease, many studies have proposed to automate the diagnosis process with the help of DL and ML. Most of the early literature in this area mainly discusses the two-class classification of breast cancer, and very few studies report on the three-class classification of using traditional manual feature extraction and machine learning. Classical feature extraction is a tedious and complex task that requires experts in the domain to work with large datasets. These methods also have some limitations in extracting the highly discriminative features from high-resolution images [32,33,34]. DL-based solutions for the early diagnosis of breast cancer made huge progress with high accuracy. In deep learning, the automatic feature learning process is carried out at different layers of CNN models, with different numbers of convolution kernels [35]. Bernard et al. [36] present a two-stage CNN-based new method for breast cancer classification into three categories. After the successful training of the first CNN model, the output of this model is input to the second CNN model for better performance. This stacked setup of CNN models produces an accuracy of 81.3%. The stacked nature of the proposed method makes it complex for the training of two CNN models.

Furthermore, with advancements in imaging technology, histopathology databases are widely utilized to automate manual detection. Sing et al. [37] proposed a TL-based method to accurately detect breast cancer, and utilized a publicly available histopathology dataset for model training. In their work, a pre-trained VGG-19 CNN is processed for training and classification purposes. A limitation of this work is the utilization of the existing CNN model without any modification. Simple transfer learning is applied, and binary classification is carried out with 90% accuracy. Roy et al. propose a study for the classification of breast cancer into binary and four categories from histology images, using a patch-level based CNN model. Their method classifies breast cancer into four categories, with an accuracy of 90%, even without utilizing the latest feature fusion and selection methods [38]. In another recent study [39], a deep learning-based modified dense network is proposed to automatically detect invasive breast cancer from histopathological images. In this study, different preprocessing tasks, such as histogram equalization and intensity normalization, are also performed to enhance the overall accuracy of the proposed model. In this work, an existing CNN model is utilized with some modifications, and only a binary classification task is performed.

Mi et al. propose a two-stage hybrid method for breast cancer multi-class classification including four types of tumors from histopathology images. In their study, 72 statistical features and the CNN were trained on different patches of the histology images for multi-classification. A multi-class accuracy of 85.19% is reported by their proposed deep learning-based method. Their two-stage method of breast cancer classification is so complex that it needs a heavy computation device [40]. In another recent work, a hybrid CNN and RNN-based method for multi-class classification of four breast cancer stages is presented. For this purpose, a pre-trained CNN model inception v3 is fine-tuned and utilized as a feature extractor. In the next step, the feature vector is utilized as input to the bidirectional LSTM for multi-class classification. The hybrid method of four-class classification produces a multi-class accuracy of 91.3% [27]. This study also contributes a large dataset of high-resolution images of four classes of breast cancer for public research purposes. The limitation of this work is that fine-tuning was only carried out on the existing CNN model, and classification is carried out without using any selection method. A transfer learning-based solution for breast cancer multi-class categorization is presented in [41]. The Alexnet pre-trained model is utilized as the base for the hierarchical model generation of multi-class classification. A feature selection method applying information gain is utilized for feature reduction purposes. For classification tasks, ML algorithms are utilized, and the highest binary accuracy of 95.48% is reported on test data. The limitation of this work is the utilization of the existing pre-trained CNN model, with a four-class classification accuracy of 92%. Sanyal et al. [42] propose a hybrid CNN model-based solution for carcinoma type categorization by using an ensemble of CNN. The ML algorithm XGBoost is utilized for carcinoma type classification, instead of the traditional softmax classifier, in order to gain high performance. This work also utilizes existing pre-trained CNN models for breast cancer classification task.

A DL-based solution for breast cancer multi-class class classification is presented in [43]. An inception-ResNet-v2-based classification framework is proposed in this work. The model training task is computed by using histopathology images that produce a multi-class accuracy of 87%. This work performed the four-class classification of breast cancer that can be further improved in terms of accuracy. In another work, Bardou et al. propose two different methods for binary and multi-class categorization of breast cancer by using histology images. In the first method, classical features are extracted using a traditional method of the bag of visual words. For the classification task, an input feature vector was provided to the SVM classifier. In their second method, a CNN model is designed and trained to automatically detect the type of breast carcinomas. Different combinations of feature sets are utilized for testing their proposed work, with the conclusion that the CNN-based method produces higher results as compared to the ML-based method. The highest multi-class average accuracy of 88% is achieved by their proposed CNN-based method, which can be further improved by utilizing a better solution [31]. The multi-class categorization of breast cancer into four classes from histology analysis by using deep learning is presented in [44]. This study proposes a feature level fusion-based method, by utilizing the inception v3 and ensemble scheme, and reports a multi-class test accuracy of 87%, which can also be further improved with a better solution. Khan et al. [45] propose the DL-based method for the same task, with a four-class classification accuracy of 88%, which can be further improved with a better solution. Wang et al. proposed a deep feature fusion-based method for breast cancer classification by using CapsNet and CNN. In their work, only binary classification of breast cancer is carried out, with an accuracy of 94% [46]. In another recent work, Agarwal et al. [47] propose a transfer learning and CNN model for breast cancer classification using the BreakHis dataset and report an accuracy of 94.6%. The limitation of their work is the binary classification of breast cancer, as multi-class classification was not performed. Gupta et al. [48] proposed a modified residual networks-based DL solution for breast cancer classification, with an accuracy of 99%, and their work is limited to only binary class classification of breast cancer. The more recent work in breast cancer classification is presented in [49,50,51,52] that can further be improved in terms of accuracy.

From the above discussion of previous methods, it is concluded that most of the work in breast cancer classification has performed the binary classification or multi-classification of four breast cancer types. The multi-class classification of breast cancer with eight types is rarely discussed, with the limitations of low classification accuracy and fixed receptive field, which can be further improved by using different approaches, such as CNN models with different receptive fields, feature selection, and feature fusion. This study proposed a deep feature fusion and selection-based method for the multi-class classification of breast cancer into eight and four classes. For this purpose, a novel 6B-Net, with six concurrent branches and each branch containing different receptive fields, is proposed. The purposed 6B-Net is implemented with six different receptive field sizes for high-level feature learning that is not found in earlier studies. The earlier studies utilized the fixed receptive field-sized convolutions that are unable to extract the highly discriminative features. This study also implemented the two nature-inspired feature selection methods ACS and PSO, and one entropy-based selection method. The purpose of using the nature-inspired feature selection method is the natural searching ability of ants and birds respectively. The entropy-based feature selection is applied to achieve faster training of the ML classifiers. Experimental outcomes show that the accuracy is significantly improved after applying selection and fusion methods. The proposed method achieves 94.20% of accuracy in breast cancer four-class classification, and 90.10% in breast cancer eight-class classification. The high performance of the proposed method in the multi-class classification of breast cancer infers that the proposed method can effectively be applied in the early detection and classification of breast cancer from histopathology images, and is a useful tool to assist pathologists in the early and accurate diagnosis of breast cancer. The limitation of the proposed method is the utilization of two publicly available datasets, but the method may also be tested on other datasets. The real-time implementation of the proposed method to specific patients was also not carried out, but this can be performed in future work. This work only tries to solve the multi-class classification of breast cancer and the segmentation of the breast cancer was not carried out, which will also be performed in future work.

## 5. Conclusions

This study proposed a deep 6B-Net with deep feature fusion and selection technique for multi-class breast cancer classification from histopathology images. For this purpose, deep features were extracted using the proposed CNN model and RESNET-50 model. After feature extraction, the feature selection methods of PSO and ACS were applied for feature selection, and horizontal feature fusion was carried out at both selected feature vectors. A feature selection method of EBS was applied before the classification task. Finally, for the multi-class classification of breast cancer different machine learning classifiers were utilized. The results were computed using the three-, five-, and ten-fold validation procedures. The highest accuracy of 90.10% for eight class classification of breast cancer was achieved with a ten-fold method, and an accuracy of 94.20% was achieved with four classes. The experimental results show that the highest accuracy is achieved with the feature fusion and selection steps. In the future, this work will be used to propose a unique feature selection method, with a novel CNN model for higher performance that also includes segmentation of the tumors.

## Figures and Tables

**Table 1:** Results using proposed feature fusion and selection-based method on BreaKHis dataset with eight classes and ten-fold validation, where Ac% represents the accuracy in percent, and T(S) represents the training time in seconds.

Classifiers	RESNET50		6B-Net		Selected		Selected		Fused		F-Selected	
	FV1		FV2		FV1		FV2		FV1 + FV2		FV1 + FV2	
	Ac%	T(s)	Ac%	T(s)	Ac%	T(s)	Ac%	T(s)	Ac%	T(s)	Ac%	T(s)
ESKNN	80.10	153	82.03	142	82.30	148	85.10	150	87.90	160	90.10	147
ESD	69.40	290	70.43	283	71.60	285	73.01	280	74.30	310	75.60	298

**Table 2:** Results using proposed feature fusion and selection-based method on BreaKHis dataset with eight classes and five-fold validation, where Ac% represents the accuracy in percent, and T(S) represents the training time in seconds.

Classifiers	RESNET50		6B-Net		Selected		Selected		Fused		F-Selected	
	FV1		FV2		FV1		FV2		FV1 + FV2		FV1 + FV2	
	Ac%	T(s)	Ac%	T(s)	Ac%	T(s)	Ac%	T(s)	Ac%	T(s)	Ac%	T(s)
ESKNN	79.11	133	81.13	125	83.32	130	84.14	132	86.90	145	90.00	128
ESD	68.40	190	70.20	175	71.60	180	71.81	185	72.30	198	73.60	181

**Table 3:** Results using proposed feature fusion and selection-based method on BreaKHis dataset with eight classes and three-fold validation, where Ac% represents the accuracy in percent, and T(S) represents the training time in seconds.

Classifiers	RESNET50		6B-Net		Selected		Selected		Fused		F-Selected	
	FV1		FV2		FV1		FV2		FV1 + FV2		FV1 + FV2	
	Ac%	T(s)	Ac%	T(s)	Ac%	T(s)	Ac%	T(s)	Ac%	T(s)	Ac%	T(s)
ESKNN	78.10	130	79.03	118	80.30	125	82.10	115	84.90	130	87.09	112
ESD	66.40	148	67.43	142	67.60	143	69.01	140	69.30	160	69.90	142

**Table 4:** Results using the proposed method on breast cancer pathology dataset with ten-fold validation, where Ac% represents the accuracy in percent, and T(S) represents the training time in seconds.

Classifiers	RESNET50		6B-Net		Selected		Selected		Fused		F-Selected	
	FV1		FV2		FV1		FV2		FV1 + FV2		FV1 + FV2	
	Ac%	T(s)	Ac%	T(s)	Ac%	T(s)	Ac%	T(s)	Ac%	T(s)	Ac%	T(s)
ESD	81.10	225	83.03	218	83.30	223	86.10	220	88.90	260	94.20	226
ESKNN	79.40	290	80.43	283	80.60	285	81.01	280	82.30	310	84.50	274
EBT	77.30	460	78.51	456	79.60	458	80.11	452	81.20	480	83.70	466
CSVM	79.55	196	80.10	190	80.18	193	81.20	191	83.16	199	85.62	188
QSVM	80.12	180	81.20	168	81.35	175	82.15	172	84.13	188	86.82	170
LSVM	80.10	123	80.13	118	81.15	125	81.99	122	83.15	135	86.11	117

**Table 5:** Results using proposed feature fusion and selection-based method on breast cancer pathology dataset with four classes and five-fold validation, where Ac% represents the accuracy in percent, and T(S) represents the training time in seconds.

Classifiers	RESNET50		6B-Net		Selected		Selected		Fused		F-Selected	
	FV1		FV2		FV1		FV2		FV1 + FV2		FV1 + FV2	
	Ac%	T(s)	Ac%	T(s)	Ac%	T(s)	Ac%	T(s)	Ac%	T(s)	Ac%	T(s)
ESD	80.10	135	82.03	130	83.00	136	85.75	132	88.10	180	93.60	136
ESKNN	78.40	249	80.13	245	80.10	242	80.88	245	82.00	270	85.0	250
EBT	76.30	218	78.00	211	79.11	215	81.00	212	81.10	225	83.00	209
CSVM	78.55	116	79.10	107	79.88	113	80.20	111	83.06	129	84.11	109
QSVM	80.12	109	81.20	103	81.01	107	81.15	105	84.01	118	85.5	103
LSVM	79.10	61	80.77	59	80.85	60	82.00	62	83.00	77	85.1	57

## Data Availability

Not applicable.

## Supplementary Materials

The following supporting information can be downloaded at: https://www.mdpi.com/article/10.3390/jpm12050683/s1: Figure S1: The sample images from the breast cancer pathology dataset that is publicly available at [27]; Table S1: The internal layers detail of the proposed 6B-Net deep CNN model; Table S2: The comparison of the proposed fusion and selection-based method with existing methods of breast cancer multi-class class classification, where Ac% represents the accuracy in percent; Table S3: The comparison of the proposed fusion and selection-based technique with existing methods of breast cancer multi-class classification, where Ac% represents the accuracy in percent.
