# Supplementary material for: Multi-Class Classification of Breast Cancer Using 6B-Net with Deep Feature Fusion and Selection Method
Source: J Pers Med. 2022 Apr 26;12(5):683. doi: 10.3390/jpm12050683 (PMC9146727; doi:10.3390/jpm12050683)
Supplement: Supplementary file 1 [file jpm-12-00683-s001.zip › jpm-1651081-supplementary.pdf]

Table S1: The internal layers detail of the proposed 6B-Net deep CNN model.

No.	Name	Feature depth	Activation	Padding	Stride	Others
**1**	Image (Input)	227x227x3	-	-	-	-
**2**	Conv1	9x9x3x128	114x114x128	Same	[2 2]	-
**3**	RL-1	-	114x114x128	-	-	-
**4**	Max_P1	-	57x57x128	Same	[2 2]	5x5 Pool size
**5**	Conv_6b-1	13x13x128x96	29x29x96	Same	[2 2]	-
**6**	Conv_6b-2	11x11x128x96	29x29x96	Same	[2 2]	-
**7**	RL-2	-	29x29x96	-	-	-
**8**	Conv_6b-3	9x9x128x96	29x29x96	Same	[2 2]	-
**9**	RL-3	-	29x29x96	-	-	-
**10**	BN-1	-	29x29x96	-	-	96 Channel
**11**	Conv_6b-4	5x5x128x96	29x29x96	Same	[2 2]	-
**12**	RL-4	-	29x29x96	-	-	-
**13**	BN-2	-	29x29x96	-	-	96 Channel
**14**	Conv_6b-5	3x3x128x96	29x29x96	Same	[2 2]	-
**15**	RL-5	-	29x29x96	-	-	-
**16**	RL-6	-	29x29x96	-	-	-
**17**	BN-3	-	29x29x96	-	-	96 Channel
**18**	BN-4	-	29x29x96	-	-	96 Channel
**19**	Conv_6b-6	7x7x128x96	29x29x96	Same	[2 2]	-
**20**	BN-5	-	29x29x96	-	-	96 Channel
**21**	RL-7	-	29x29x96	-	-	-
**22**	BN-6	-	29x29x96	-	-	96 Channel
**23**	Add-1	-	29x29x96	-	-	-
**24**	RL-8	-	29x29x96	-	-	-
**25**	Gmax_p1	-	1x1x96	-	-	-
**26**	Grp_Conv1	3x3x1x96	1x1x9216	Same	[1 1]	-
**27**	RL-9	-	1x1x9216	-	-	-
**28**	Max_P2	-	1x1x9216	Same	[1 1]	5x5 Pool size
**29**	Drop1	-	1x1x9216	-	-	50% Drop
**30**	FC_1	-	1x1x4096	-	-	-
**31**	RL-10	-	1x1x4096	-	-	-
**32**	Drop2	-	1x1x4096	-	-	50% Drop
**33**	FC_2	-	1x1x100	-	-	-
**34**	SMax1	-	1x1x100	-	-	-
**35**	Classification	-	-	-	-	Cross entropy

Table S2: The comparison of the proposed fusion and selection-based method with existing methods of breast cancer *multiclass class classification,* where Ac% is representing the accuracy in *percent.*

Reference	Year	No. of Classes	Ac%
**Albashish et al. [28]**	2021	08	89.83
**Karthiga and Narashimhan [29]**	2021	08	89.29
**Rao PMM et al. [30]**	2021	08	89.00
**Bardou et al. [31]**	2018	08	88.23
**Proposed method**		08	90.10

Table S3: The comparison of the proposed fusion and selection-based technique with existing methods of breast cancer *multiclass classification,* where Ac% is representing the accuracy in *percent.*

Reference	Year	No. of Classes	Ac%
**Yan et al. [27]**	2020	04	91.30
**Golatkar et al. [49]**	2018	04	85.00
**Cao et al. [50]**	2018	04	87.00
**Mi et al. [40]**	2021	04	85.19
**Zorgoni et al. [51]**	2021	04	93.75
**Ibraheem et al. [52]**	2022	02	91.37
**Proposed method**		04	94.20

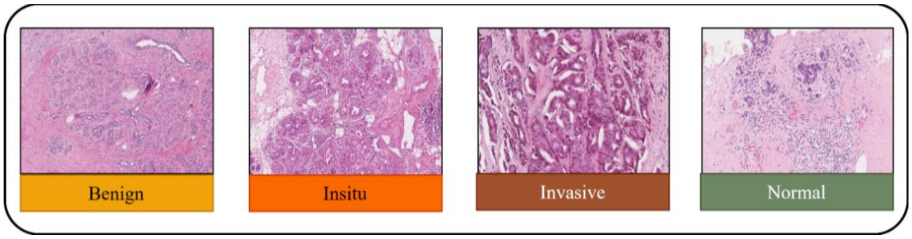

Figure S1: The sample images from breast cancer *Pathology Dataset that are publicly available at* [27].
